# Air Pollution Coverage, Anti-Chinese Sentiment, and Attitudes Towards Foreign Policy in South Korea

**DOI:** 10.1007/s11366-023-09849-z

**Published:** 2023-03-27

**Authors:** Esther E. Song

**Affiliations:** German Institute for Global and Area Studies, Institute for Asian Studies, Rothenbaumchaussee 32, 20148 Hamburg, Germany

**Keywords:** Sino-Korea relations, foreign policy attitudes,, environmental pollution, public opinion

## Abstract

Air pollutants allegedly originating from China have become a thorny issue in South Korea. Despite a neutral view of the topic on the part of the South Korean government, recent public polls show a high correlation between the air pollution issue and negative sentiment toward China. How has the media reported on China regarding air pollutants in South Korea? What is the effect of media reports on air pollution on anti-Chinese sentiment and foreign policy attitudes? By examining news headlines and Twitter data in 2015 and 2018, this work finds that media reports blaming China for air pollution doubled during the 2015–2018 period. Discourse surrounding air pollution also shifted: negative sentiment directed at both the Chinese government and the Chinese people increased in 2018 compared to 2015. In addition, an original online survey experiment shows that China-blaming articles have a causal effect on increasing related resentment, particularly toward Chinese people, and that this effect is moderated by age group. Such articles have also had negative effects on foreign policy attitudes via increased anti-Chinese sentiment; greater hostility toward the Chinese people is found to have a causal effect on reduced support for strengthening relations with their country.

**Supplementary Information:**

The online version contains supplementary material available at 10.1007/s11366-023-09849-z.

## Introduction

In 2022, China-South Korea relations entered their 30th anniversary. The two countries have enjoyed increased economic and cultural exchange since the establishment of these ties in 1992. Since 2004, China has been South Korea’s biggest trading partner, with a volume thereof reaching USD 0.14 trillion in 2021 [36]. Chinese immigration to South Korea makes up the largest proportion of such arrivals, accounting for 43.6% [38]. Aside from economic partnership, China also plays an important role in determining peace in the peninsular region by having the greatest degree of leverage over North Korea.

This article covers a relatively new but seldom examined issue that has become a central topic informing anti-Chinese sentiment in South Korea since the mid-2010s: air pollution. Although it is known that the South Korean public’s attitude toward China worsened due to the Northeast Project (*dongbukgongjeong*) in 2004 and the Terminal High Altitude Missile Defense (THAAD) crisis in 2016, more recently air pollution has become the China-related issue with the highest negative ratings among the country’s citizens. For example, according to a survey conducted by SisaIn in April 2021, the top reason for an unfavorable view of China is air pollution [39]. The latter has become a key topic impacting South Korea-China relations since the mid-2010s due to increased controversy over the source of this pollution. While promising stronger measures against China to the domestic audience, the South Korean government has taken a mild stance on the issue, merely requesting cooperation from the Chinese government. For instance President Moon Jae-in, during his presidential race in 2017, mentioned that he would “discuss the matter of air pollutants in the China-Korea summit” [30], being later criticized for failing to take a stronger position regarding the issue.

A backlash from civil society then ensued: the Air Pollutant Informant (*misemeonji allimi*) [1], a Facebook group created in April 2017, has been dedicated to attributing blame to the Chinese government and criticizing South Korean domestic experts who advocate for shared responsibilities between the two countries. In March 2018, an online petition was filed on the Blue House website that demanded the South Korean government seek resolution from its Chinese counterpart, with it receiving 270,000 signatures [3]. In response, both parties agreed to establish the China-Korea Environmental Cooperation Center [14]. Yet in 2019, Lu Kang, the spokesperson for the Ministry of Foreign Affairs in China, denied that the latter was the source of such pollution [68], sparking public protests by conservative groups in front of the Chinese Embassy in Seoul [35].^1^

Regarding the increased anti-Chinese sentiment circulating among the South Korean public, some have raised the question of whether the media has exacerbated such animosity: in 2019, Li Ganjie, head of the Ministry of Ecology and Environment in China, told Cho Myung-rae, head of the Ministry of Environment in South Korea, that the latter’s media exaggerates the role of China in contributing to air pollution in South Korea [31]. Likewise, previous works have shown that media outlets reporting on air pollution are biased against China [34, 55]. Inspired by these discussions, this article examines how the media has reported on China and domestic air pollution in South Korea, and whether this has contributed to increasing anti-Chinese sentiment and less benign foreign policy attitudes. Despite the importance of this topic, there is currently no work that empirically examines the link between reporting patterns regarding air pollution and anti-Chinese sentiment, or the implications of this for views on the two countries’ relations. By combining text analyses of social media data and a survey experiment, the causal link between media reporting on air pollution, anti-Chinese sentiment, and foreign policy attitudes is thus examined.

The findings hereof contribute to the growing literature highlighting the intersection between the media, public opinion, and policymaking [43, 62]. Regarding foreign policy, the International Relations scholarship has discussed how public opinion can affect related decisions: it has been found that in democracies, leaders can be punished through elections and therefore they care about public opinion when it comes to foreign policy [45, 57]. Extant works have noted that the media acts as a “conveyor belt” of information and frames an event in ways consistent with elite rhetoric [7, 70]. The media thus acts as a passive channel transmitting elite views to the general public, and so helps harden the latter’s attitudes in line with those of the state. However, a growing body of literature has also divulged that the media can play a proactive role in shaping public opinion in ways impacting foreign policy too. Entman showed that the media is likely to have an independent influence on foreign policy when leaders debate the frames for a given course of action [17]. More recently, the proactive role of the media in influencing foreign policy has been highlighted in relation to the rise of new media outlets [6]. By examining how China’s contribution to air pollution in South Korea has been reported on, and the effects of this on anti-Chinese sentiment, this article contributes to the burgeoning scholarship emphasizing the proactive role of the media in shaping foreign policy attitudes.

This is also broadly connected with the increasing work on xenophobia and its consequences. Recent studies have found that governments, media, and conspiracy theories can create narratives targeting specific groups of people, inciting xenophobic sentiment [27, 42, 52]—as also linked to nationalism [22, 67]. Such resentment has repercussions for various government preferences, including vis-à-vis immigration [21], foreign policy [41], and trade [37]. In the context of South Korea, anti-Chinese sentiment has been relatively understudied compared to anti-Japanese or anti-American views, which have already been found to have strong effects on foreign policy [13, 49, 53]. The scholarship has viewed anti-Chinese sentiment in South Korea as weaker than its anti-Japanese counterpart due to different historical experiences with the countries involved [15]. Yet due to the aggravation of anti-Chinese sentiment in recent years, reaching an all-time high in 2022 [2, 60], the phenomenon has begun to attract greater academic attention—particularly in relation to the 2022 presidential elections as well to the security implications of such resentment [58, 59, 64].

The use of social media data to examine trends in anti-Chinese sentiment in South Korea has both its advantages and its limitations. For example, regarding the latter, there is the issue of representativeness: while results vary by country, the consensus is that Twitter users are younger (for the United Kingdom, see [46]; for the United States, see [8]; for South Korea, see [25]). Yet, there are also benefits to be had in using online- versus survey data to examine anti-Chinese sentiment: the digital environment provides opportunities for freer speech, which makes it easier to detect the phenomenon in question. Due to the anonymity the Internet affords, people can say things they might otherwise not do so face-to-face [61]. This tendency has also been found to be stronger regarding hate speech and racism—anonymity can embolden people to be more outrageous, obnoxious, and antagonistic in what they say [9, 16, 54]. Based on this, I utilize both social media- and survey data to gauge anti-Chinese sentiment. First, I use social media data to show descriptively the trends herein associated with the air pollution issue; second, I test the causal effect of China-blaming reporting on anti-Chinese sentiment and less benign foreign policy attitudes.

The article is organized as follows. First, I describe how public sentiment toward China in South Korea has changed over time since 1992. Feelings had been positive in the early 1990s but would gradually worsen due to China’s indulging of North Korea and coercive diplomacy surrounding the THAAD missile base. In more recent periods, the issue of cross-border air pollution has become a top contributing factor to high levels of resentment toward China among South Korean citizens. Second, I analyze media coverage of air pollution in the 2015–2018 period and show how articles blaming China for it increased year on year. Compared to 2015, such articles had doubled by 2017. Third, I analyze Twitter data to show how the South Korean public discourse regarding air pollution and China would evolve: by 2018 hostility directed at the Chinese government and the Chinese people had emerged, having not featured in 2015. Lastly, I show results from a unique online survey experiment testing the causal effect of China-blaming articles on anti-Chinese sentiment, and subsequently on how the resentment aroused here has affected foreign policy attitudes.

## Changing Public Perceptions of China in South Korea

In 1992, when China and South Korea first established official relations, public perceptions in the latter were positive due to increased expectations that the former would play a positive role in realizing Korean unification. China then ranked as the second-most favorable neighboring country, surpassing Japan [10, 63]. In 1996, the percentage of respondents in one study agreeing that China is an important ally to South Korea was more than double those believing so for the US (47.1 versus 23%) [19]. This all aligns with the South Korean government’s expectation that diplomatic ties with China would enable denuclearization and peaceful unification. This positive outlook was also embedded in official Ministry of Foreign Affairs documents released regarding the rapprochement initiated with China in 1992 to these two ends [47].

The turning point came in 2004 however, when China launched the Northeast Project that created controversy among South Korean politicians and was covered widely in the domestic media. The Northeast Project was highly contentious due to its alleged politicization of history and negation of sovereignty. Thus, China was framed as a hostile superpower having goals of “Chinese expansionism” (*junghwapaegwonjuui*) in the South Korean media. Public perceptions also worsened: between 2004 and 2006, respondents with a favorable view of China decreased from 49 to 32% [30]. The trade dispute arising over South Korea’s restriction of garlic imports from China and the subsequent retaliation from the latter also contributed to negative feelings here [29].

Attitudes toward China further worsened throughout the 2010s, due to increased concerns over the country’s role in failing to contain threats from North Korea—which became a widely covered topic due to a series of attacks by the latter on South Korea, including the shelling of Yeonpyeong Island and the sinking of the Cheonam patrol corvette in 2010. Throughout the second decade of the new century such perceptions continued to decline, reaching their lowest point in 2013 when just 23% of respondents in one study reported having a favorable view of China—a 26% decrease therein from 2004 [18]. In 2016, the THAAD crisis—a foreign policy dispute that emerged over the establishment of a missile basis in Seongju, South Korea—raised a series of concerns over China’s coercive diplomacy. Negative perceptions of China were also reflected in contemporary polls, with unfavorable views thereof increasing from 37% to 2015 to 60% in 2018 [60]—representing a 23% increase in just three years.

Although the number of works on the causes of anti-Chinese sentiment in South Korea is small compared to those on anti-Japanese views due to South Korea’s different historical experiences with the two countries [15], a growing trend can still be detected here—with it having a particular focus on nationalism and domestic polarization, and specific attention being given to the Northeast Project and the THAAD disputes. Some scholars have focused on nationalism: it was found that the historical dispute over the ancient kingdom of Koguryo has had a causal effect on anti-Chinese sentiment [20]. Domestic polarization and media reporting have also received scrutiny: for example it has been also found that, compared to the Northeast Project issue, domestic polarization was higher regarding THAAD, where South Korea’s right-wing parties and conservative media used rhetoric designed to weaken support for the incumbent government’s pro-China policy and to strengthen a pro-US stance instead [28].

Meanwhile, the issue of air pollution has begun to rank higher here than these two disputes in recent years. According to a survey by SisaIn, the top reason for negative sentiment toward China was air pollution (89.4%), followed by COVID-19 (86.9%) [39]. In the same survey, over 94% of respondents answered that China is responsible for South Korea’s domestic air pollution. Although the origins of the latter are a scientific issue, the question has been raised in both politics and scholarship as to whether the media is playing a role in the heightened belief that air pollution can be directly attributed to China. The South Korean government has remained neutral on the issue: when President Moon met Yang Jiechi, member of the Politburo of the Chinese Communist Party, during a meeting in March 2018, he mentioned that “there are domestic causes for air pollution in Korea, but also partially from China”—thus framing the issue as a joint one [32]. Yet when examining the strong backlash from civil society, as reflected in the aforementioned online petition uploaded to the Blue House website, there are strong indications that the media has indeed played a role in steering blame toward China. In the petition it says: “The media also reports that the cause of air pollution originates from China, yet the government is irresponsive.” Media reports are cited as evidence. The petition also asks: “Why do we need to cooperate? Air pollutants are from China” [3, 5]. Previous works on the South Korean media’s reporting vis-à-vis air pollution in the country have also found that responsibility for it is attributed to China alone: blaming the latter has emerged as one of four frames used, and can be found in both liberal and conservative outlets alike [55]. Furthermore, most articles that have mentioned the causes of air pollution have attributed blame to China, as opposed to domestic or individual behavioral factors [34]. These findings raise several questions: How exactly has the media directed blame toward China for air pollution in South Korea? How has the public discourse regarding Chinese responsibility for such pollution changed over time? Does this attribution affect anti-Chinese sentiment and negative perceptions of the two countries’ relations? I turn to these matters in the following.

## Media Coverage of Air Pollution and China-Blaming: 2015–2018

To examine the patterns in air pollution reporting and how they might have fueled anti-Chinese sentiment, headlines from the largest news aggregator in South Korea, Naver News, were crawled (https://news.naver.com/). I scoured the top-five search results for each day from 2015 to 2018 after searching the term “China,” and then filtered out news articles that mentioned “fine dust air pollutants.” This yielded 145 articles from 75 unique sources.^2^ Other than collecting data from individual news sources, reliance is on Naver News for several reasons. The key one, however, is that it has been found that most South Korean citizens consume such information through search sites and news aggregators [51]. According to recent research, 72% of respondents said that they use search engines and news aggregators to consume news. This is much higher than the proportion (5%) stating that they consume news directly from related websites, and hence my choice here.

**Fig. 1:**
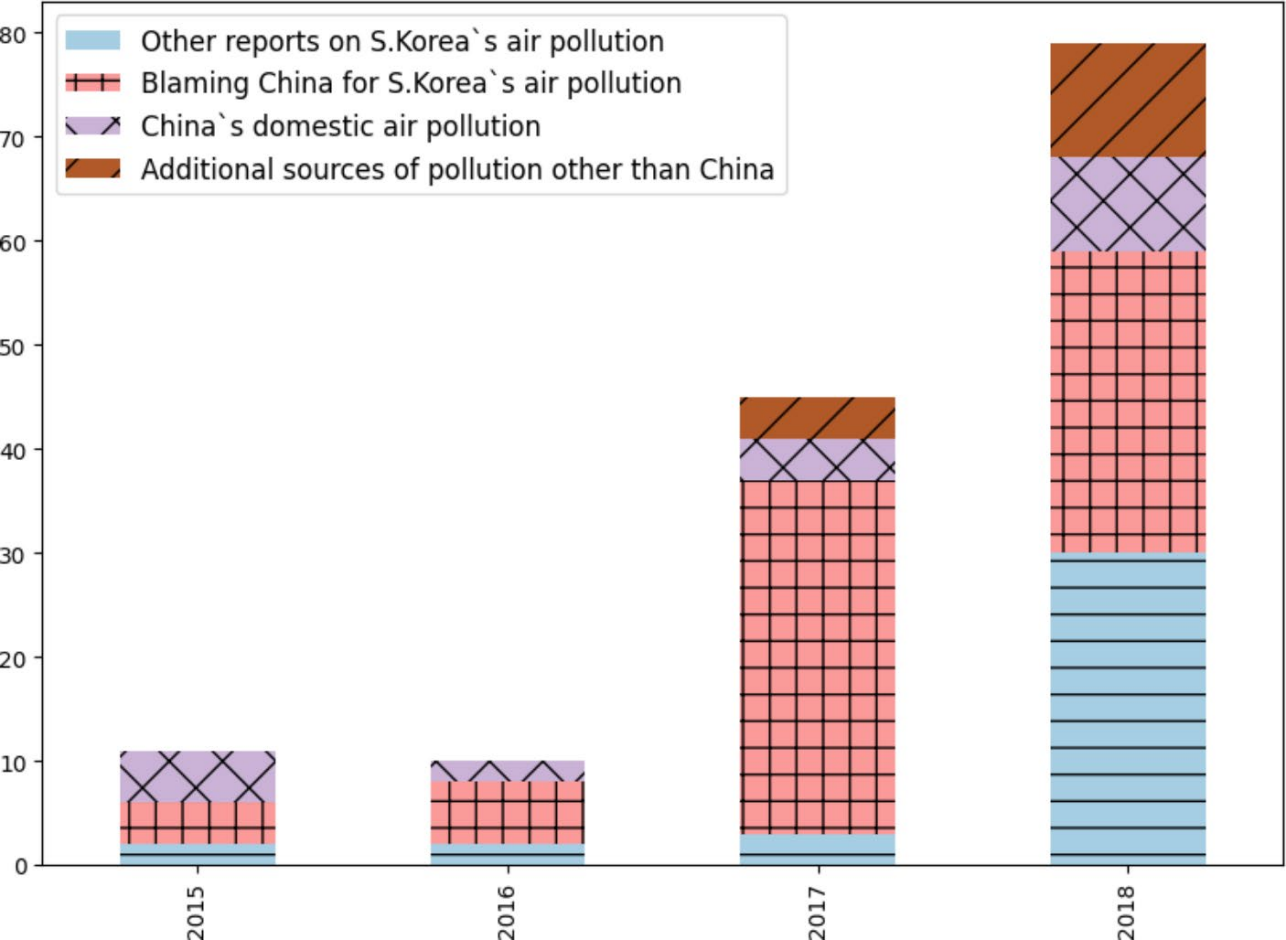
Categorization of news on air pollution linked to China (2015–2018)

Media reports linking China to fine dust air pollutants increased fourfold between 2015 and 2017 (see Fig. 1 below). Not only did the total number of related articles increase, but the proportion of those blaming China for South Korea’s air quality also went up twofold—from approximately 36% in 2015 to 75% in 2017. These articles included a phrase that directly attributes blame to China for this air pollution: “fine dust air pollutants from China” (*junggukbal misemeonji*). At the same time, while accounting for a smaller proportion than the China-blaming articles, there was also an increase in ones mentioning additional sources of pollution (such as domestic factors); these went from none in 2016 to approximately 13% doing so in 2018. Another category that grew in size was articles reporting on South Korea’s air pollution issue, with them mushrooming in 2018. The two categories seem to have grown due to the online petition posted on the Blue House website in 2018 urging China to take greater responsibility, as well as following South Korean government efforts to increase cooperative measures with the latter [3, 5]. Lastly, articles discussing the state of China’s domestic air pollution grew slightly during the 2017–2018 period, but the proportion remained small. This included not only reports on worsening air pollution within China but also on the latter’s efforts to tackle it [48, 50]. Table 1 below shows example headlines for each category of report established.

**Table 1:** Categorization of News on Air Pollution and China (N = 145)

Categorization	News Headlines
Articles that blame China for S.Korea’s air pollution	“Fine dust air pollutants at high levels across the country, ‘the cause is China’” (*Korea Economic Daily*, 2015) “Fine dust air pollutants at high due to smog from China” (*Newsis*, 2017) “Sudden attack by fine dust air pollutants from China…high levels during this weekend” (*SBS*, 2017) “Huge smog from China…fine dust air pollutants make it hard to breathe” (*SBS*, 2018)
Articles that mention additional sources of pollution other than China	“The source of horrific fine dust air pollutants is not only China” (*OhmyNews*, 2017) “Are the fine-grained air pollutants from China? Probably not this time” (*Hankyoreh*, 2017) “China’s contribution was small for January’s fine dust air pollutants…around 38∼57%” (*Yeonhap News*, 2018) “Recent fine dust air pollution can be attributed more to domestic causes” (*Yeonhap News*, 2018)
Articles on China’s domestic air pollution	“China announces red alert…fine dust air pollutants 20 times more than safety baseline” *Newsway*, 2015) “Liaoning province starts managing air quality in a serious manner” (*Yeonhap News*, 2017)
Other reports on S.Korea’s air pollution	“POSCO ICT exports dust collector to China” (*Yeonhap News*, 2016) “Ministry of Environment reports that it will increase cooperation with China in order to reduce air pollutants” (*Yeonhap News*, 2018)

The statistics show that reports on air pollution and those attributing blame to China would heavily increase in the post-2016 period. Against this backdrop, a key question arises: Have these articles fueled anti-Chinese sentiment, and, if so, how has the pattern changed over time? To answer this, I first examine anti-Chinese sentiment related to air pollution in Twitter data from 2015 to 2018.

## Topic Modeling of Tweets: 2015 and 2018

To answer the question of how public sentiment changed toward China amid increasing media reports attributing blame to the latter for South Korea’s air pollution, I draw on related tweets from 2015 to 2018 respectively. These data were used to delineate changes in the public discourse after the increased coverage of China and more hostile rhetoric seen from 2017 onward. For comparison, I collected all tweets for 2015 and 2018 by searching the keyword “China” and “fine dust pollutants.” I avoided using tweets from 2016 to 2017 due to the strong salience of the THAAD dispute in those years, which made it difficult to sample tweets related to China and air pollution. As media coverage of THAAD dissipated after South Korea and China resolved to halt further expansion of the missile base in November 2017, I chose the period after that year as the point of comparison with the earlier one of 2015.

The data were collected using an open-source Python package “snscrape,” which enables the amassing of historical tweets (archived at: https://github.com/JustAnotherArchivist/snscrape).^3^ As a result, the 2015 sample included 401 tweets; for 2018, the total number collected was 2,951. The research method used was topic modeling. Specifically, I used the Non-Matrix Factorization (NMF) model to detect topics in the tweet corpus. NMF is an unsupervised method that categorizes text corpora without labels. In addition, compared to other topic models such as Latent Dirichlet Allocation (LDA), it has been found that NMF performs better when dealing with shorter texts. While LDA assumes that each text in the sample discusses several topics, NMF does not—making the latter more applicable to shorter, single-issue texts [12].

Text preprocessing, such as removing punctuation, duplicates, and emojis, was conducted prior to applying the NMF model. In addition, I removed any tweets related to weather reports and advertisements so that the sample only includes ones posted by individuals. I used the KoNLPy (Korean NLP in Python; https://konlpy.org/en/latest/) open Korean Text module to tokenize tweets to nouns. I chose term frequency and inverse document frequency (TF-IDF) vectorization. Compared to the count vectorizer (the bag-of-words approach, which counts the frequency of unique words that appear in a string), TF-IDF notes how many times a unique word occurs across all strings—therefore effectively ignoring common words.

## Results: Anti-Chinese Sentiment Linked to Air Pollution Increased in 2018

The optimal number of topics for the respective text corpora for 2015 and 2018 was found by obtaining the coherence score [56]. Developed in studies of natural language processing, this score evaluates whether each output topic is coherent on a scale of 0 to 1. According to the coherence score, the optimal number of topics for the 2015 corpus was 5; for 2018, meanwhile, it was 15. In both periods, the number of tweets spiked when pollution levels increased: when examining changes in the number of tweets in each topic category, the number surged around March and April in both years, when pollution levels spiked, and then decreased during the summer, when pollution rates decreased. The number of tweets spiked again in November and December, when pollution levels increased. Illustrations of these trends are provided in the Appendix (Figures A1, A2).

There are similarities and differences between the topics extracted from the 2015 versus 2018 corpora. Among those remaining similar throughout, one comprised a discussion of Asian dust that usually occurs in spring (Topic 1 in Table 2; Topic 6 in Table 3 below); another was complaints about fireworks being the cause of the air pollution coming from China (Topic 3 in Table 2; Topic 4 in Table 3). At the same time, there was a sharp difference between the topics obtained from the two periods: compared to 2015, tweets conveying anger toward China and the Chinese people were prevalent throughout 2018. These “venting” tweets often included racist or otherwise derogatory remarks targeted at the Chinese people (Topics 3 and 10 in Table 3). The examples here also reveal that the primary cause of the increase in such racist and derogatory remarks was blaming China for South Korea’s air pollution. Another difference is that in 2015 there were more discussions on the actual origins of fine dust pollutants (Topics 2 and 5 in Table 2), while in the 2018 corpus this inquiry did not emerge. Further, in 2018 more grievances were expressed toward the South Korean government for its reluctance to resolve the issue with China, which seems to have been driven by the controversy arising from the online petition posted on the Blue House website calling for China to be held responsibility and stronger government efforts from the latter to tackle the issue (Topic 14 in Table 3).

**Table 2:** NMF Topic Analysis Results for 2015 Tweet, Selected Topics Only (N = 401)

Topic Number	Topic	Summary	Sample Tweets (English)
1	Asian dust, mask, wind, government, because, really, MERS, we, countermeasures, regulations	Linking annual dust to China and the wind; comments on how the first MERS patient in China was Korean; asking government countermeasures and regulatory efforts	‘China creates dust wind with fine dust air pollutants’ ‘Korea reciprocated by sending MERS patient and received fine dust air pollutants in exchange, looks like fair trade’ ‘How is the government regulating companies to reduce fine dust air pollutants? Since when has fine dust air pollution become so severe and how did the government regulate or de-regulate companies? The government just thought it was from China.’
2	Korea, emerged, right now, extent, itself, air, conclusion, talk, MERS	Discussion over the actual origins of fine dust air pollutants; venting over anti-Korean sentiment in China over a Korean MERS patient	‘In fact some of the fine dust air pollutants are from China but a lot of them are also emitted domestically a lot of people blame China when we talk about decreasing air pollution however a lot of it comes from automobiles and coal factories however when I say these things facebook users get angry and tell me to blame China’ ‘actual air quality around car road are worse than publicly announced indicators the cause of fine dust air pollutants are not coal factories or China but are automobiles on the street’ ‘so China sends over Asian dust and the Chinese people commits all kinds of crime including chopping people up or phone scamming and Chinese people are criticizing Koreans for sending over MERS sounds like they like damaging people but they hate when others do it to them let’s just spread MERS to this ch*** country’
3	Today, density, Seoul, mask, fireworks, smog, dust, reason, Chinese New Year, yesterday	Discussion over whether fine dust air pollutants are due to fireworks in China during Chinese New Year	‘Is it really true that fine air pollutants are from fireworks used during the Chinese New Year in China is that a direct cause’ ‘I heard today’s air pollution is because of all the fireworks during Chinese New Year’
5	Korea, we, emerged, country, think, people, environment, half, continue, problem	Discussion over whether the origins of fine dust air pollutants are from Korea, discussions that both sides share responsibility	‘fine dust air pollutants…half of it is actually from Korea so we have equal responsibility with China’ ‘China is about % responsible for the fine dust air pollutions in Korea over half originates from within the major causes are gas emissions from diesel automobiles and smoke from thermal power stations’

**Table 3:** *NMF Topic Analysis Results for 2018 Tweet, Selected Topics Only (N = 2,951)*

Topic Number	Topic	Summary	Sample Tweets (English)
1	Really, f***ing, weather, please, pain, smog, protest, earth, have done, school	Complaints about smog and air pollution and anger venting towards China; hoping more days off from school and companies due to bad air quality	‘air quality is really bad today so if we can’t blame China then let’s have some days off school and work’ ‘I want to really get rid of China this fine dust is so annoying people don’t take it seriously when we call it dust this is more like cancer-inducing smog’
3	F***, f***ing, son of a b****, county, reason, bulls***, heat wave, this, c****, oneself	Anger over air pollution and attributing blame to China; venting anti-Chinese sentiment	‘air quality is so s***** today China f***ing a********.’ ‘there is so much rain today but tomorrow’s forecast says air is super bad what the f*** China’
4	Because, fireworks, respiratory, sound, thermal power plant, face, definitely, sky, how, Chinese New Year	Complaints over fireworks during Chinese New Year in mainland as cause of air pollution; complaints and assertions that air pollution is due to China	‘f*** heard all this fine dust is because fireworks in China’ ‘fine dust is definitely because of China is not because of thermal power plants or automobiles that’s all bulls****’ ‘so everyone knows now that all this fine dust comes from China they can’t get away with it give me back my throat and nose’
6	Asian dust, tomorrow, one, citizen, now, smog, measures, afternoon, really, about	Complaints over Asian dust and how it originates from China, blaming China; expressing hatred towards China by attributing blame for Asian dust and smog	‘f*** it is so hard to breathe under this mask because of all the Chinese fine dust this feels like s***’ ‘in spring it’s the Asian dust in summer there’s the heat wave in fall there is fine dust and winter there is serious smog China m***********’
10	S***, c****, Chinese people, numbers, bulls***, heat wave, problem, always, see, commute	Blaming China for the heat wave and air pollution; venting ager towards the Chinese people for fine dust air pollutants	‘China f****** gives us fine dust and heat waves’ ‘of course these c**** f***** will not like hearing it but we are suffering for months because air pollution and it feels like s*** the only nice Chinese are the dead ones’
14	Domestic, problem, solve, element, government, measures, emerged, citizen, voice, effect	Complaints on South Korean government for being irresponsive and unaccountable regarding air pollution issues, how it has been reluctant in safeguarding citizen health	‘Moon Jae-in is a disaster he can’t say anything to the Chinese communist party while people feel threatened because of the cancer-inducing fine dust why can’t he demand apology from China like he did to Japan why can’t he demand compensation’ ‘I started off with talking about why can’t Moon say anything to China about fine dust problem is it because of the rice price spikes and coals in North Korea wonder what the hell is in his head is it only North Korea and commies who the hell voted this guy I said this and that guy from Soon-cheon said all the rest are red necks who should I vote for so I lost my words’

## Online Survey Experiment

### Data and Hypotheses

Although Twitter data show that there would be an increase in anti-Chinese sentiment coinciding with the greater number of articles appearing in the media blaming China for the air pollution issue, they do not confirm whether this actually affected people’s perceptions of the country. Social media data have limits in terms of inference: although they offer advantages in exposing hate speech, they lack representativeness. Specifically, Twitter users are younger on average, which suggests that the trend observed above could be limited to a sample of youth citizens. In addition, selection bias might exist—those expressing negative feelings toward China online might already have strong anti-Chinese tendencies. To disentangle these problems of endogeneity and representativeness, I conducted an online survey experiment to test the causal effect of China-blaming articles on anti-Chinese sentiment. In addition, I tested whether such articles have impacted foreign policy attitudes by fomenting resentment here.

The online survey experiment was conducted with 500 South Korean adults aged 20–70 years old and was carried out in March 2022 by Hankook Research, a public opinion research company in South Korea. The sample was randomly drawn from the company’s survey panel. The experiment was conducted by briefly presenting respondents with a news article, which was randomly shown to the participant. This comprised either: (1) a news item attributing blame to China and including the phrase “air pollutants from China” in the headline or (2) a news item that remained neutral on the issue. Also, following the finding that the majority of the South Korean population consumes news via online aggregator sites, only a short length of time (of minimum 10 s) was required for the respondents to be exposed to the treatment or control before they moved on to subsequent questions. Those presented after the treatment or control were offered randomly to minimize satisficing. The treatment (1) and control (2) details are reported in Table 4. Summary statistics are presented in the Appendix (Table A1). The total number of respondents was 504, and the size of the treatment (N = 253) and control (N = 251) groups was almost even. The distribution of covariates such as age, education, income, and gender were similar between the two groups.

**Table 4:** Treatment and Control for the Survey Experiment

	Treatment	Control
Title	Fine dust levels “bad” all day, due to smog from China remaining inland	Fine dust levels 2–3 times higher than usual – “bad” until tomorrow morning
Text	Fine dust pollutants from China covers the whole country, and the fine dust rating is “bad”. According to the Korean Environment Corporation, the average fine dust level in Seoul at 7 am this morning is 101*μ*g/m^3^, which is more than twice of annual average fine dust level (50*μ*g/m^3^). This level is equivalent to air quality level within car tunnels. Aside from Seoul, fine dust levels in other areas are: Choong-book, Jeonbook, Daejeon, Daegu, Gwangju, Busan, which are all high levels. A staff at the National Institute of Environmental Research said that “fine dust levels will be low during the afternoon but will increase sometime later in the afternoon”. The reason why fine dust level is “bad” these days is because the smog from China remains within the South Korean inlands.	Air pollutants and fog have been creating smog for four days. The fine dust levels have increased 2–3 times than usual, and the smog will continue until tomorrow morning. The fog decreased during the day time, but the pollutants remained creating hazy sky view. Fine dust levels have increased 2–3 times than usual, and the levels are at “bad” level. PM2.5 particles, which are smaller than 2.5 micrometers consist of 70–80% of the entire fine dust mass. Since these small particles can enter lungs, the elderly and patients with respiratory disease should refrain from being outside. National Institute of Environmental Research analyzed the cause that pollutants created within the country and those that came from China were mixed, and since the air circulation lessened, the pollutants have accumulated in the air.

The sampled respondents were randomly split; the treatment group was primed with a news item that blamed China for air pollution, and the control group was primed with a news item that was neutral in terms of reporting the causes hereof. The survey included questions that captured individual covariates such as age, gender, and education level. Questions were included on one’s feelings regarding the Chinese government and the Chinese people respectively: (1) How favorably do you feel toward China? (2) How favorably do you feel toward the Chinese people? The response was aggregated from a 5-point Likert scale to a 3-point Likert scale (3=“Unfavorable,” 2=“Neutral,” 1=“Favorable”) due to the low response counts mapping to “Very Favorable” and “Favorable” on the 5-point Likert scale.^4^ These two separate dimensions were considered due to the finding in the literature that anti-Chinese sentiment consists of negative attitudes toward the country’s both government and people [4].

To capture the dependent variable, questions on foreign policy attitudes and perceptions of China were added to the survey. More specifically, these asked respondents about their view on the rise of China and whether South Korea should strengthen relations with that country amid heightened US-China hegemonic competition. These two topics were included due to recent survey results showing that the top two issues South Koreans perceive as threatening national interests in the future are (1) the rise of China and (2) greater US-China hegemonic competition [33].^5^ The question relating to the first dimension asked whether respondents agreed with the following statements: (1) the rise of China will become a serious military threat to South Korea, (2) the rise of China will become a serious economic threat to South Korea. Responses were captured on a 5-point Likert scale and then aggregated to a 3-point Likert scale. The question concerning the second dimension, meanwhile, was constructed as: (3) What do you think South Korea should do regarding increasing US-China rivalry in Northeast Asia? Three response options were given: (a) “Strengthen relations with the United States,” (b) “Maintain balance,” or, (c) “Strengthen relations with China.” In the subsequent analysis, I created a dichotomous variable of 1=“Strengthen relations with China” and 0=“Other” (“Maintain balance” or “Strengthen relations with the United States”) to estimate the causal effect of intensified anti-Chinese sentiment on the South Korean populace’s foreign policy attitudes.

One source of bias potentially arising during the analysis was that respondents may have been strongly interested in the issue of air pollution and thus might have sought more information on the phenomenon. This would have potentially exposed them to a greater number of articles blaming China for the latter. This increased exposure could have led to selection bias, where respondents were more likely to harbor negative feelings toward China. A question on one’s interest in air pollution was included to control for this: “How interested are you in air pollution?” Responses were 5=“Very interested,” 4=“Interested,” 3=“Neutral,” 2=“Disinterested,” and 1=“Very disinterested.” This variable was also later aggregated to a 3-point Likert scale prior to the analysis.

As this online survey experiment was devised to test whether exposure to news articles that attribute blame to China for air pollution in South Korea has had an actual effect on inciting anti-Chinese sentiment among the latter’s populace, the following hypothesis was formulated:

H1: Exposure to news blaming China for domestic air pollution has increased anti-Chinese sentiment.

To test whether exposure to China-blaming articles has led to negative foreign policy attitudes via an increase in anti-Chinese sentiment, a second hypothesis was then formulated:

H2: Exposure to news blaming China for domestic air pollution has had negative effects on foreign policy attitudes via anti-Chinese sentiment.

*Analysis of H1: Exposure to Articles Blaming China for Air Pollution has Increased Anti-Chinese Sentiment, Particularly toward the Chinese People, and the Effect Is Moderated by Age Group*.

Because the dependent variable of sentiment (toward the Chinese government and Chinese people respectively) was categorical (1=“Favorable,” 2=“Neutral,” 3=“Unfavorable”) and ordinal, I used ordinal logistic regression to test H1. The independent variable is a dummy one indicating treatment and control (1 = Treatment, 0 = Control). I also included the interaction term between age and treatment to account for recent findings that anti-Chinese sentiment varies across age groups [58].

**Fig. 2:**
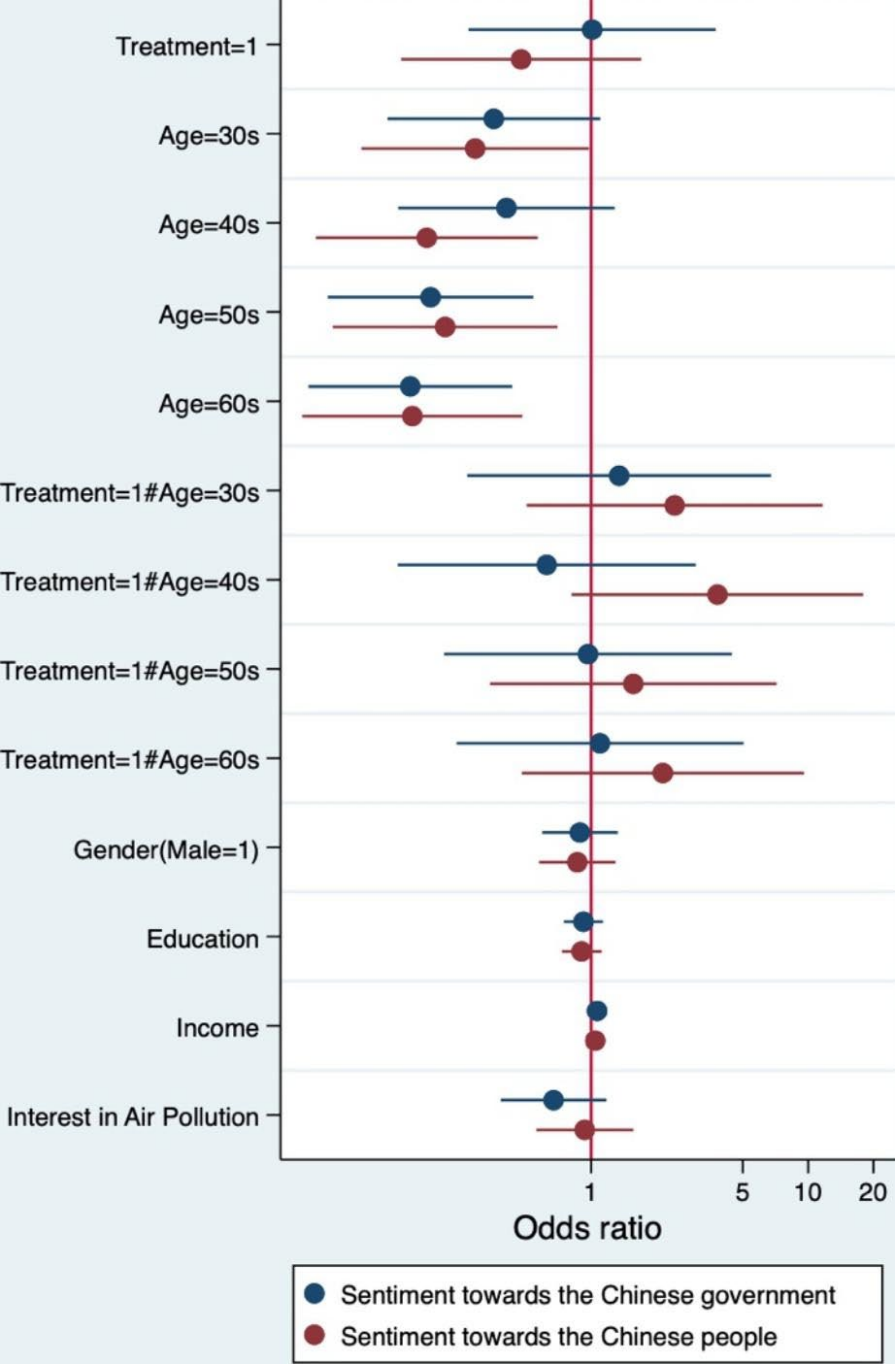
Ordinal logistic regression results (Hypothesis 1, odds ratio)

Table 5; Fig. 2 above report the results of the log-odds from the ordinal logistic regression. They indicate some support for H1—although the treatment itself did not have a statistically significant effect on the outcome variables, the interaction term between treatment and age, especially for the 40–49 age group, was statistically significant at the 10% confidence level. The estimated log-odds suggest that online survey respondents were 3.8 times more likely to dislike the Chinese people when they were exposed to a China-blaming article and when they were in their 40s compared to those in the baseline group—those who were treated and were in their 20s. In addition, the effect of the treatment on the respective age groups was stronger for sentiment toward the Chinese people than for that toward the Chinese government. Figure 2 shows that the exponentiated coefficient estimates of the interaction term are larger than 1 for all those between 30 and 69 years old when the dependent variable is sentiment toward the Chinese people rather than toward the Chinese government. In short, those falling within this age range were more likely to dislike the Chinese people when they were treated with an article blaming that country for South Korea’s air pollution compared to those aged 20–29, whereas there was no such effect when the dependent variable was instead sentiment toward the Chinese government.

**Table 5:** *Ordinal Logistic Regression Results, Log-Odds (Hypothesis 1)*

Dependent Variable	Sentiment towards Chinese government (3 = Unfavourable 2 = Neutral 1 = Favourable)	Sentiment towards Chinese people (3 = Unfavourable 2 = Neutral 1 = Favourable)
	Model I	Model II
Treatment	1.010	0.475
	(0.02)	(-1.14)
Gender	0.887	0.863
	(-0.58)	(-0.71)
Age = 30s (Baseline = 20s)	0.356*	0.292**
	(-1.80)	(-2.00)
Age = 40s	0.407	0.175***
	(-1.53)	(-2.91)
Age = 50s	0.182***	0.212**
	(-3.06)	(-2.55)
Age = 60s	0.147***	0.150***
	(-3.48)	(-3.18)
Education	0.921	0.904
	(-0.77)	(-0.95)
Income	1.065	1.045
	(1.43)	(0.99)
Interest in Pollution (1 = Not Interested; 2 = Neutral; 3 = Interested)	0.671	0.933
	(-1.40)	(-0.26)
Treatment#30s	1.345	2.426
	(0.36)	(1.11)
Treatment#40s	0.624	3.820*
	(-0.58)	(1.70)
Treatment#50s	0.967	1.564
	(-0.04)	(0.58)
Treatment#60s	1.098	2.140
	(0.12)	(0.99)
N	504	504

*Note.* Exponentiated coefficients; *t* statistics in parentheses

^*^p < 0.10, ^**^p < 0.05, ^***^p < 0.01

*Analysis for H2: China-Blaming Articles Decreases Support for Strengthening Relations with China via Increasing Anti-Chinese Sentiment*.

Several steps were taken before testing H2. First, although the question is a binary classification one, the data were heavily imbalanced, where those in favor of strengthening relations with China accounted for only 1.5% of the entire pool of online survey respondents. In a classification question, this could result in lack of statistical power when applying the model. To correct this imbalance, I used SMOTENC (synthetic minority oversampling technique for nominal and continuous [11]), which allows for correcting imbalances in both numerical and nonnumerical data. Compared to the classic oversampling technique, which duplicates data points from the minority class, SMOTENC uses a k-nearest-neighbor algorithm to create synthetic data. First, random data from the minority class are chosen, then k-nearest neighbors from the datasets selected. Synthetic data that lie between the random data and the randomly selected k-nearest neighbor are now created. In this study, the corrected imbalance ratio was 9%, and the total number of data points equaled 550 (Appendix, Table A2).

I used ordinal logistic regression to estimate the effects of anti-Chinese sentiment on three dependent variables regarding foreign policy attitudes: (1) the perception of China as a military threat; (2) the perception of China as an economic threat; and, (3) attitudes toward strengthening relations with China. For the third, I used logistic regression because the variable was binary (1=“Strengthen relations with China,” 0=“Other”). Results for Models III and VI in Table 6 below show that there is a strong negative correlation between the treatment and the alliance choice. The results in Model II show that when a person was treated with an article blaming China for South Korea’s air pollution, the odds of that respondent being in favor of strengthening relations with China decreased by 95%, and this effect was statistically significant at the 1% confidence level.

**Table 6:** *Ordinal Logistic Regression & Logistic Regression Results, Log-Odds (Hypothesis 2)*

	IV: Sentiment towards Chinese government			IV: Sentiment towards Chinese people		
DV	(I) China will be a serious military threat	(II) China will be a serious economic threat	(III) Strengthen relations with China	(IV) China will be a serious military threat	(V) China will be a serious economic threat	(VI) Strengthen relations with China
Sentiment towards Chinese government	1.795^**^	1.605^**^	0.548^**^			
	(2.34)	(2.50)	(-2.38)			
Sentiment towards Chinese people				2.001^***^	1.433^*^	0.124^***^
				(2.81)	(1.83)	(-5.94)
Treatment	0.914	0.966	0.045^***^	0.949	0.983	0.035^***^
	(-0.29)	(-0.15)	(-4.19)	(-0.17)	(-0.08)	(-4.22)
Gender	0.705	0.758	0.0193^***^	0.708	0.758	0.0140^***^
	(-1.11)	(-1.24)	(-3.86)	(-1.09)	(-1.25)	(-3.99)
Age	1.250^*^	1.202^**^	1.382^**^	1.242^*^	1.173^**^	1.238
	(1.85)	(2.22)	(2.24)	(1.82)	(1.97)	(1.24)
Income	1.147^*^	1.016	0.923	1.144^*^	1.018	0.979
	(1.92)	(0.35)	(-0.96)	(1.87)	(0.38)	(-0.22)
Education	0.669^**^	0.858	1.446^**^	0.684^**^	0.860	1.348
	(-2.46)	(-1.36)	(1.97)	(-2.32)	(-1.34)	(1.48)
*N*	504	504	550	504	504	550

*Note.* Exponentiated coefficients; *t* statistics in parentheses

^*^p < 0.10, ^**^p < 0.05, ^***^p < 0.01

Although there was a high correlation between the treatment and the alliance choice, the chosen model specifications did not test whether the treatment affected the alliance choice via anti-Chinese sentiment. The logistic regression specification tested correlation rather than causation in this regard. To further examine this link, I used the causal mediation algorithm developed by Tingley et al. [65]. The estimation process consists of two steps: First, fitting the model that predicts the mediating variables, which here are: (a) sentiment toward the Chinese government and (b) sentiment toward the Chinese people. Second, estimating a model that predicts the dependent variable—support for foreign policy that strengthens relations with China—using treatment variables, the mediating variables, and all other covariates included in the first step. Then, the average direct effect (ADE) and the average causal mediation effect (ACME) are estimated. ADE captures the effect of the treatment variable on the outcome one less the effect via the mediating variable, while ACME captures the effect of the treatment on the outcome via the mediating variable [26]. The analysis was conducted using mediation package in R [65]. Because the treatment was only statistically significant when the dependent variable was strengthening relations with China (Models III and VI), and not the perception of China as a military and economic threat, I examined whether anti-Chinese sentiment mediated the causal effect of treatment on alliance choice.

Table 7 shows that anti-Chinese sentiment—particularly hostility toward the Chinese people—had a causal effect on attitudes toward foreign policy. ACME revealed that the mediating variable—anti-Chinese sentiment—impacted the outcome variable of support for strengthening relations with China. The second column in Table 7 shows that this effect was statistically significant at the 10% level. However, the effect was not statistically significant in the case of negative sentiment toward the Chinese government. For both mediating variables, under ADE the direct effect of the treatment on the outcome aside from the mediating variable was statistically significant, which implies that there were other unobservable factors affecting alliance choice via the treatment variable besides anti-Chinese sentiment.

**Table 7:** *Causal mediation analysis results (p-values)*

	Mediator: sentiment towards the Chinese government	Mediator: sentiment towards the Chinese people
ACME (average)	0.78	0.076 *
ADE (average)	< 2e-16 ***	< 2e-16 ***
Total Effects	< 2e-16 ***	< 2e-16 ***

*Note.* Sample Size Used: 550. Simulations: 500

^*^p < 0.10, ^**^p < 0.05, ^***^p < 0.01

## Discussion

The controversy that has arisen over the source of South Korea’s air pollution has become one of the strongest correlates to anti-Chinese sentiment in the country in recent years. How has South Korea’s media reported on air pollution, and how has this contributed to increased anti-Chinese sentiment there? How has this coverage affected the domestic population’s foreign policy attitudes? By examining how the South Korean media has reported on air pollution, this study showed that blame for it was increasingly attributed to China during the 2015–2018 period. It was revealed how this had the negative causal effect of creating unfavorable sentiment toward China—particularly toward the Chinese people. Moreover, the article found that this heightened resentment has had a detrimental impact on South Koreans’ foreign policy outlooks—especially regarding strengthening China-South Korea relations.

The findings of the paper both demand discussion and leave room for further research. First, although the correlation between media bias against China and anti-Chinese sentiment are understandable as a high degree of association was found between these aspects both in Twitter data (2015 and 2018) and in the online survey experiment, it is not clear why the effect of that media bias was stronger among respondents in the 40–49 age group. One possible explanation for this is the heuristics generated by the China-blaming articles—lack of information on government responsibility and the overt framing of where the air pollution comes from through the phrase “fine dust pollutants from China” could have primed individuals to attribute blame to the Chinese people for emissions as opposed to the Chinese government, which has a responsibility for managing pollution.

Previous research has shown that media heuristics matter in terms of who people attribute blame to for public policy failures, and that they tend to take onboard the apportioning of responsibility laid out in the media [23, 66]. In addition, it has been found that specifying public officials’ culpability here by revealing their titles affects the ways in which the public blames these individuals for said policy failures [44]. In the context of South Korea, partisanship could also be in play considering that people in their 40s are the strongest supporters of the Democratic Party [69] and that the Moon administration’s foreign policy was largely pro-China. Thus, the media heuristics could have pushed respondents in their 40s to attribute blame more to the Chinese people than to the Chinese government. Future work should further empirically examine whether variation in blame attribution vis-à-vis the Chinese people correlates with exposure to information pertaining to the Chinese government’s role in regulating pollution levels, and how this is mediated by partisanship.

Regarding the link between air pollution, anti-Chinese sentiment, and foreign policy attitudes, one possible explanation for the nature of it could come from image theory. The latter stipulates that countries can be considered as being held to an image, such as an “enemy,” “ally,” or “imperialist” [24]. The increased hostility toward China in relation to the air pollution issue may reflect how the South Korean public sees it as an enemy, an actor that could potentially pose a threat, reducing their will to cooperate (i.e. to strengthen alliances/relations). Future research should thus examine whether this negative causal effect on foreign policy preferences is moderated by particular sets of images and threat perceptions. Lastly, this work has limited external validity; despite the low salience of the air pollution issue in the presidential elections that took place in March 2022, it was still covered in preceding debates. This could have served to heighten the public’s overall awareness of the issue. As the online survey took place after these presidential elections, that increased familiarity may have contributed to the results obtained—albeit with less impact on the results’ internal validity.

## Electronic Supplementary Material

Below is the link to the electronic supplementary material.

Supplementary Material 1

## Footnotes

1 These groups included Freedom Union (*jayuyeondae*), Freedom Korea National Defense Corps (*jayudaehan hogukdan*), and Turn Right. Far-right groups and the Liberty Korea Party (LKP, later merged into the People’s Power Party) used anti-China slogans as part of protests against the South Korean government in 2018 and 2019. For example, in a large-scale protest waged by the LKP in 2019, slogans included “Anti-China & Pro-U.S.,” while the focus of the rally was largely anti-President Moon [40]. Such anti-China slogans were also part of protests focused on criticizing the Moon administration’s foreign policy, which leaned toward being pro-China.

2 Articles from liberal outlets accounted for approximately 12% hereof, while those from conservative outlets accounted for 3%. Liberal outlets refer to the following: *Kyunghyang Shinmun*, Nocut News, Newsis, Pressian, and *Hankyoreh*. The remaining outlets were categorized as neutral. Conservative outlets were: TV Chosun, Chosun Biz, *Chosun Ilbo*, *Joongang Ilbo*, Channel A, *Donga Ilbo*, and *Hankook Gyeongjae*.

3 As this package collects only publicly available data on Twitter, Institutional Review Board approval was not obtained for the collection procedure.

4 “Very favorable” (2) and “Favorable” (23) accounted for 5% of the total responses. To construct the 3-point Likert scale, the responses “Very unfavorable” and “unfavorable” were also merged.

5 According to the fourth round of a survey on Korean identity in May 2020, carried out by the East Asia Institute, the top issue that South Koreans perceived as threatening to the national interest was the rise of China as a great power (41.7%), followed by US-China hegemonic competition (34.9%) [33].
