# Supplementary material for: Air Pollution Coverage, Anti-Chinese Sentiment, and Attitudes Towards Foreign Policy in South Korea
Source: J Chin Polit Sci. 2023 Mar 27:1–22. Online ahead of print. doi: 10.1007/s11366-023-09849-z (PMC10040909; doi:10.1007/s11366-023-09849-z)
Supplement: Supplementary file 1 — Supplementary Material 1 [file 11366_2023_9849_MOESM1_ESM.docx]

**Appendix**

# **Figure A1**

# Topic Distribution and Average PM10 Levels, 2015


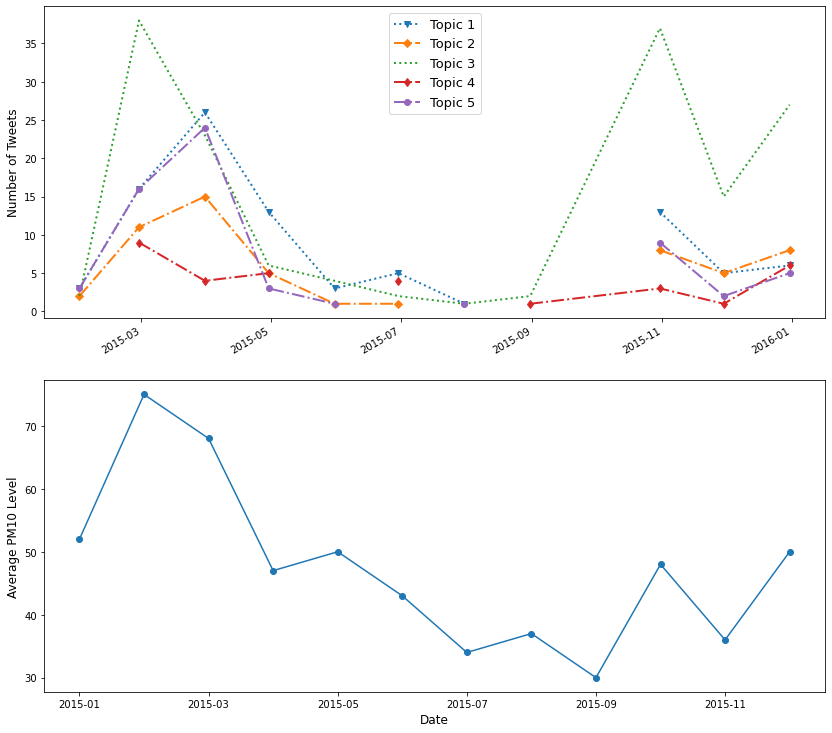


# **Figure A2**

# Topic Distribution and Average PM10 levels, 2018


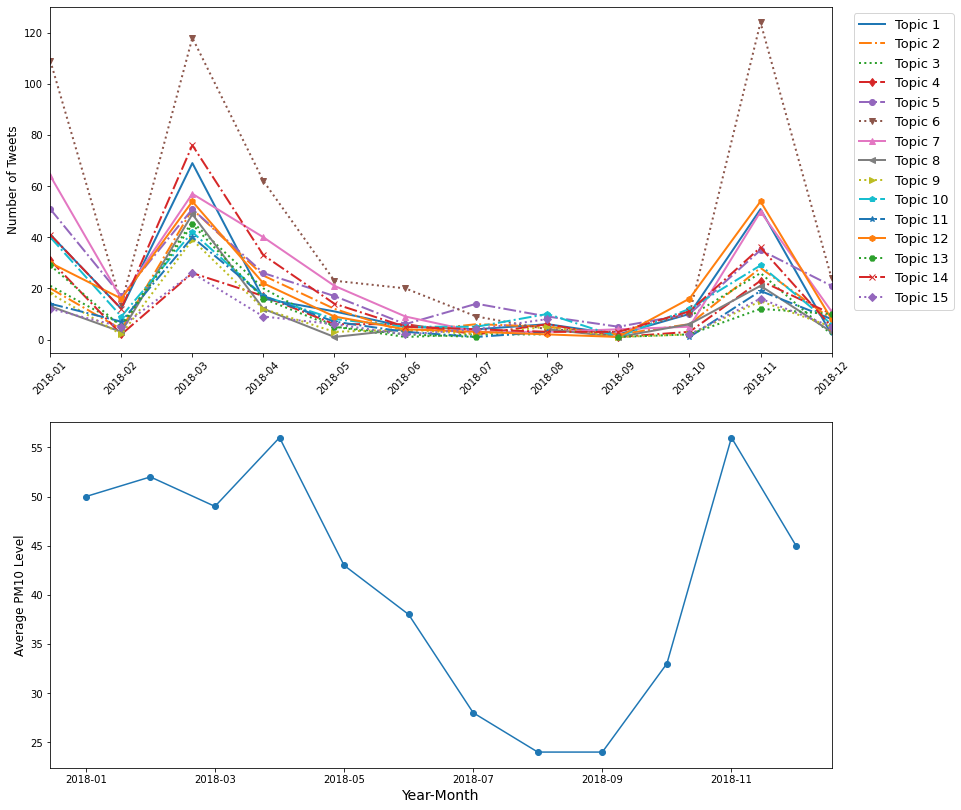


**Table A1**

Summary Statistics (N=504)

|  | **(1)** |  |  |  | **(2)** |  |  |  |
| --- | --- | --- | --- | --- | --- | --- | --- | --- |
|  | **Control** | | | | **Treatment** | | | |
|  | **Mean** | **SD** | **Min** | **Max** | **Mean** | **SD** | **Min** | **Max** |
| Sentiment towards Chinese government (3=Unfavourable 2=Neutral 1=Favourable) | 2.652 | 0.609 | 1.000 | 3.000 | 2.673 | 0.526 | 1.000 | 3.000 |
| Sentiment towards Chinese people (3=Unfavourable 2=Neutral 1=Favourable) | 2.692 | 0.527 | 1.000 | 3.000 | 2.689 | 0.550 | 1.000 | 3.000 |
| Gender (Male=1) | 0.502 | 0.501 | 0.000 | 1.000 | 0.506 | 0.501 | 0.000 | 1.000 |
| Age | 3.016 | 1.411 | 1.000 | 5.000 | 3.000 | 1.414 | 1.000 | 5.000 |
| Education | 4.455 | 1.048 | 1.000 | 7.000 | 4.382 | 0.982 | 2.000 | 7.000 |
| Income | 6.209 | 2.428 | 1.000 | 12.000 | 6.275 | 2.577 | 1.000 | 12.000 |
| Interest in Air Pollution (3=Interested 2=Neutral 1=Disinterested) | 2.798 | 0.483 | 1.000 | 3.000 | 2.849 | 0.381 | 1.000 | 3.000 |
| N | 253 |  |  |  | 251 |  |  |  |

**Table A2**

Summary Statistics (Imbalance Corrected, N=550)

|  | **(1)** |  |  |  | **(2)** |  |  |  |
| --- | --- | --- | --- | --- | --- | --- | --- | --- |
|  | **Control** | | | | **Treatment** | | | |
|  | **Mean** | **SD** | **Min** | **Max** | **Mean** | **SD** | **Min** | **Max** |
| Sentiment towards Chinese government (3=Unfavourable 2=Neutral 1=Favourable) | 2.615 | 0.638 | 1.000 | 3.000 | 2.673 | 0.526 | 1.000 | 3.000 |
| Sentiment towards Chinese people (3=Unfavourable 2=Neutral 1=Favorable) | 2.588 | 0.587 | 1.000 | 3.000 | 2.693 | 0.548 | 1.000 | 3.000 |
| Gender | 0.432 | 0.496 | 0.000 | 1.000 | 0.504 | 0.501 | 0.000 | 1.000 |
| Age | 3.081 | 1.351 | 1.000 | 5.000 | 3.000 | 1.417 | 1.000 | 5.000 |
| Education | 4.510 | 1.025 | 1.000 | 7.000 | 4.382 | 0.982 | 2.000 | 7.000 |
| Income | 6.135 | 2.293 | 1.000 | 12.000 | 6.272 | 2.605 | 1.000 | 12.000 |
| Interest in Air Pollution (3=Interested 2=Neutral 1=Disinterested) | 2.824 | 0.454 | 1.000 | 3.000 | 2.850 | 0.379 | 1.000 | 3.000 |
| Alliance Choice (1=Strengthen relations with China 0=Maintain balance or strengthen relations with U.S.) | 0.162 | 0.369 | 0.000 | 1.000 | 0.008 | 0.089 | 0.000 | 1.000 |
| N | 296 |  |  |  | 254 |  |  |  |
